# Supplementary material for: Interaction of Thymine DNA Glycosylase with Oxidised 5-Methyl-cytosines in Their Amino- and Imino-Forms
Source: Molecules. 2021 Sep 22;26(19):5728. doi: 10.3390/molecules26195728 (PMC8510025; doi:10.3390/molecules26195728)
Supplement: Supplementary file 1 [file molecules-26-05728-s001.zip › molecules-1345339-supplementary.pdf]

Supplementary Information for Interaction of Thymine DNA Glycosylase with Oxidised 5-Methyl-Cytosines in their Amino- and Imino-Forms

Senta Volkenandt[1,2,‡], Frank Beierlein[2,‡], and Petra Imhof[1,2,*]

31st August 2021

[1]Department of Physics
Freie Universität Berlin
Arnimallee 14
14095 Berlin

[2]Department for Chemistry and Pharmacy
Computer Chemistry Centre
Friedrich-Alexander University (FAU) Erlangen Nürnberg
Nägelsbachstrasse 25
91052 Erlangen

‡These authors contributed equally.
*petra.imhof@fau.de

DNA conformation

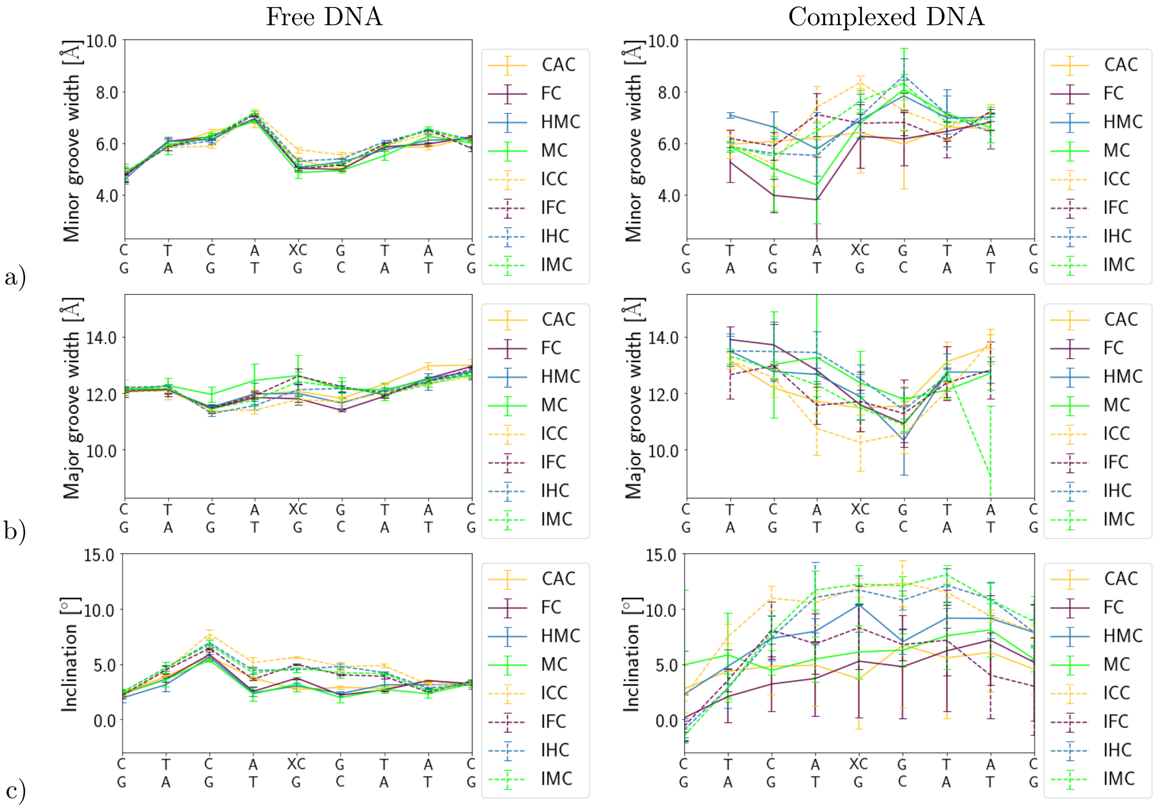

Figure S1: DNA parameters a) major groove widths b) minor groove widths, and c) inclination. The parameters shown on the left hand side are calculated from the simulations of the free DNA, those on the right hand side are calculated from the simulations of the complexed DNA.

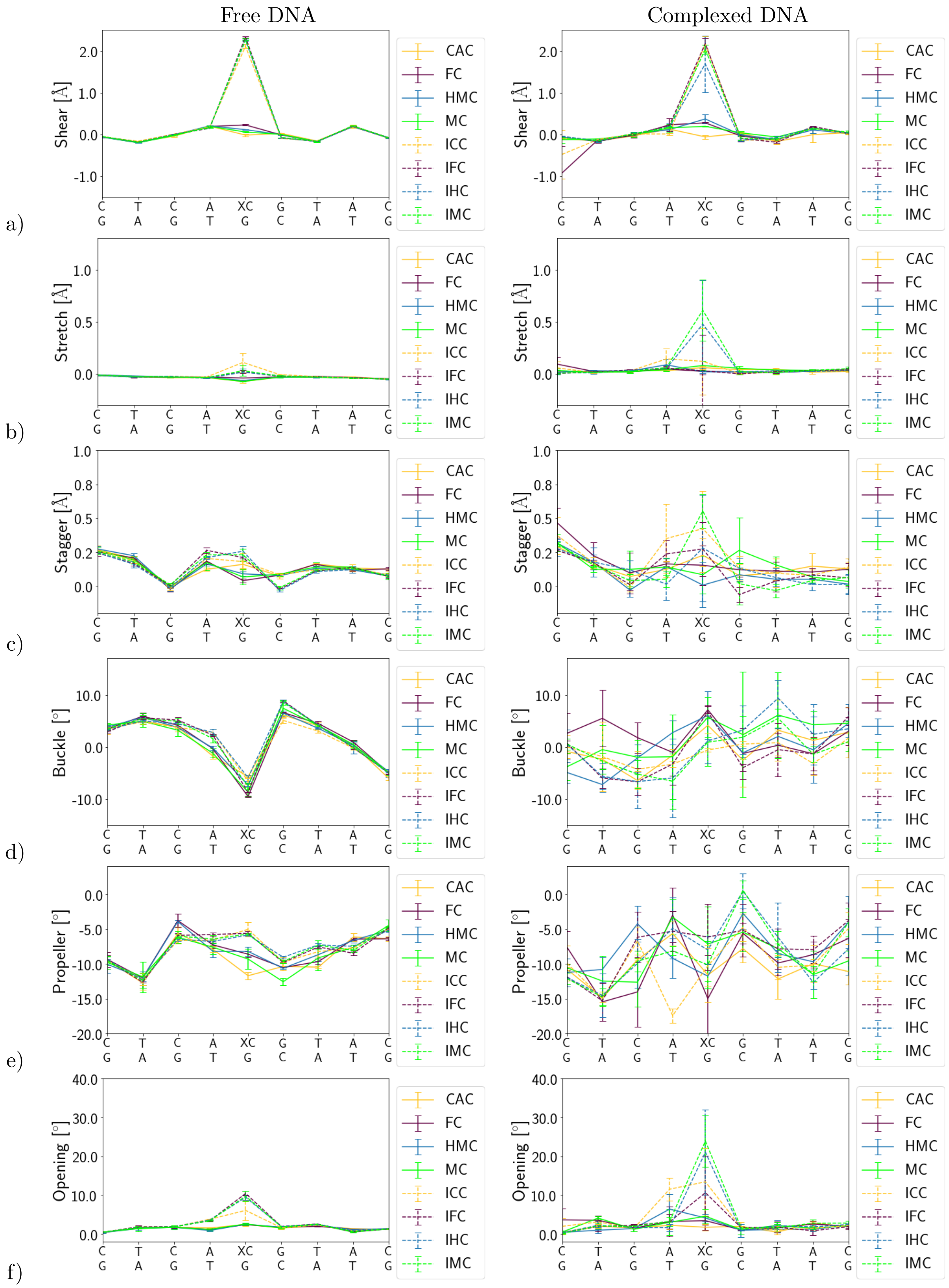

Figure S2: DNA base pair parameters a) shear b) stretch, c) stagger, d) buckle, e) propeller, and f) opening angle. The parameters shown on the left hand side are calculated from the simulations of the free DNA, those on the right hand side are calculated from the simulations of the complexed DNA.

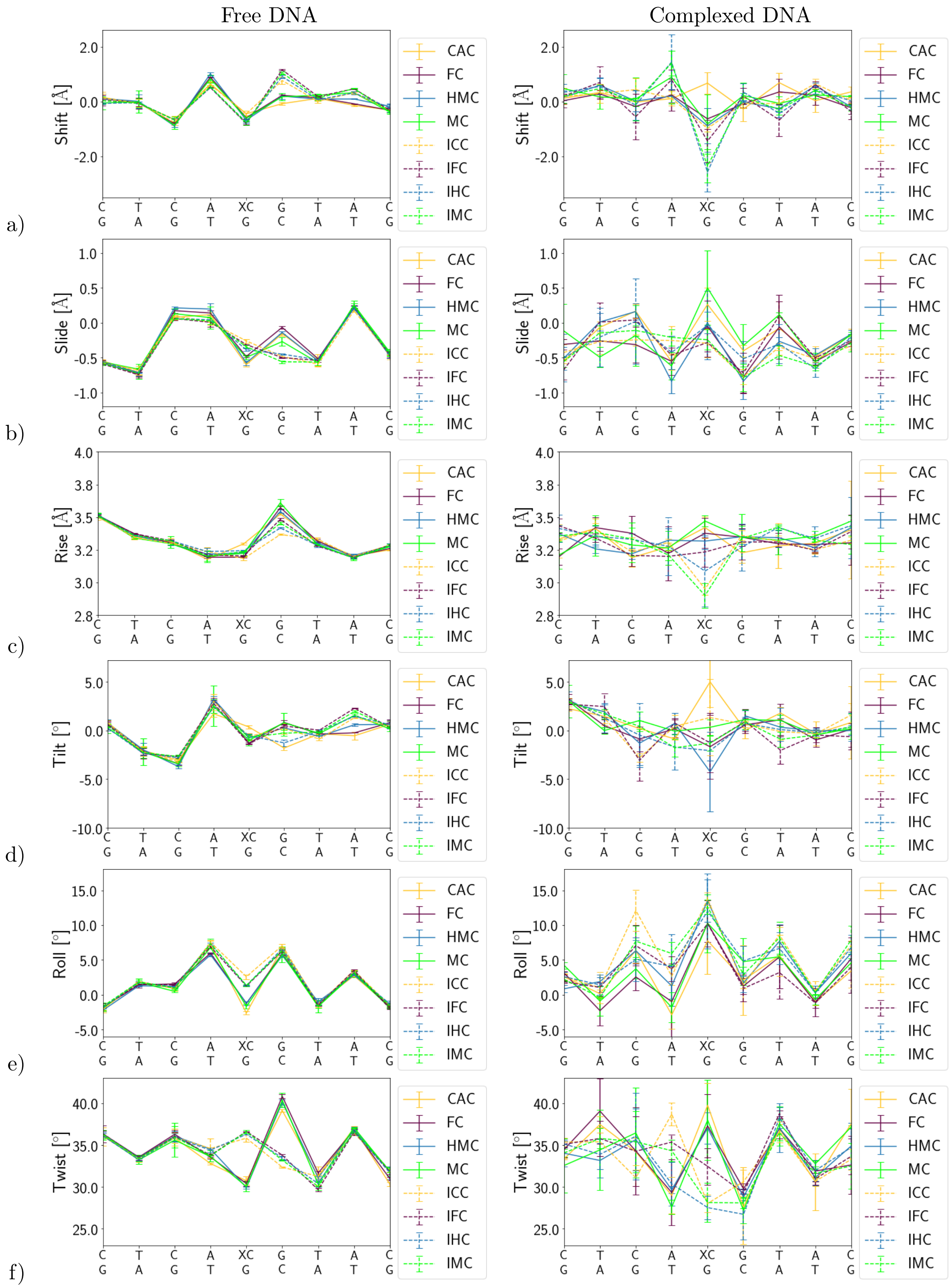

Figure S3: DNA base step parameters a) shift b) slide, c) rise, d) tilt, e) roll, and f) twist. The parameters shown on the left hand side are calculated from the simulations of the free DNA, those on the right hand side are calculated from the simulations of the complexed DNA.

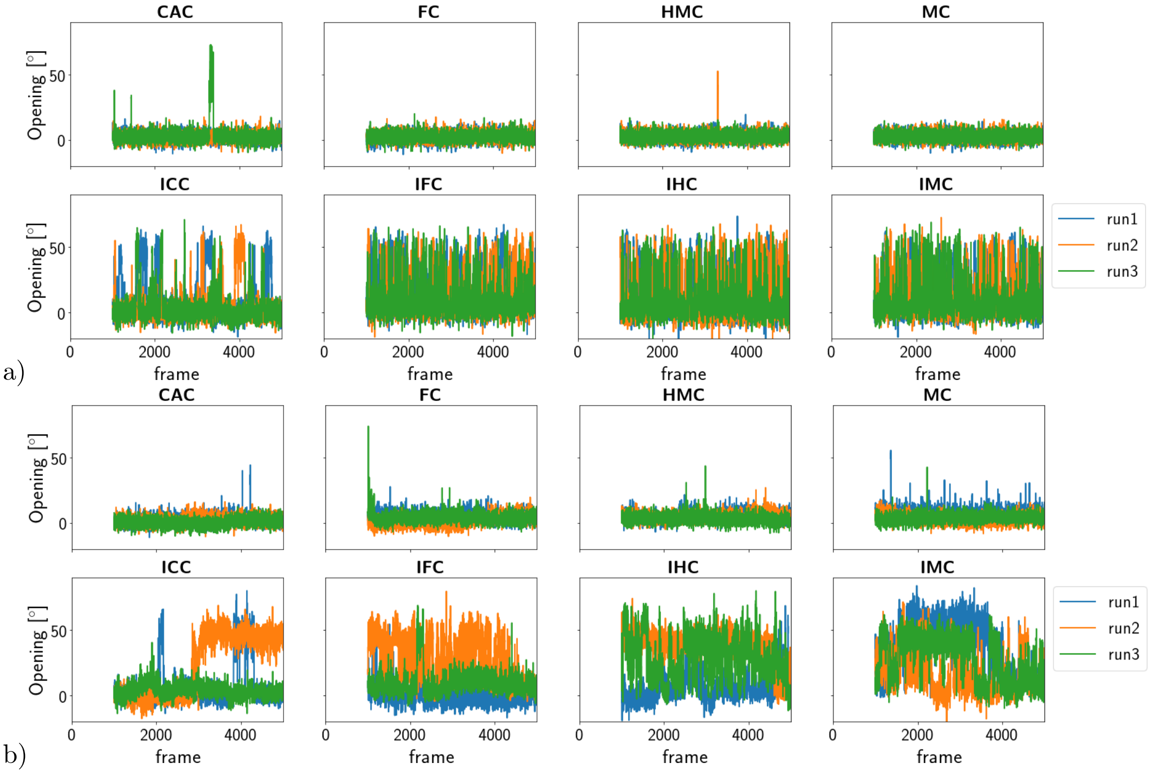

Figure S4: Time series of the opening angle in the individual runs of a) free DNA and b) DNA complexed to TDG.

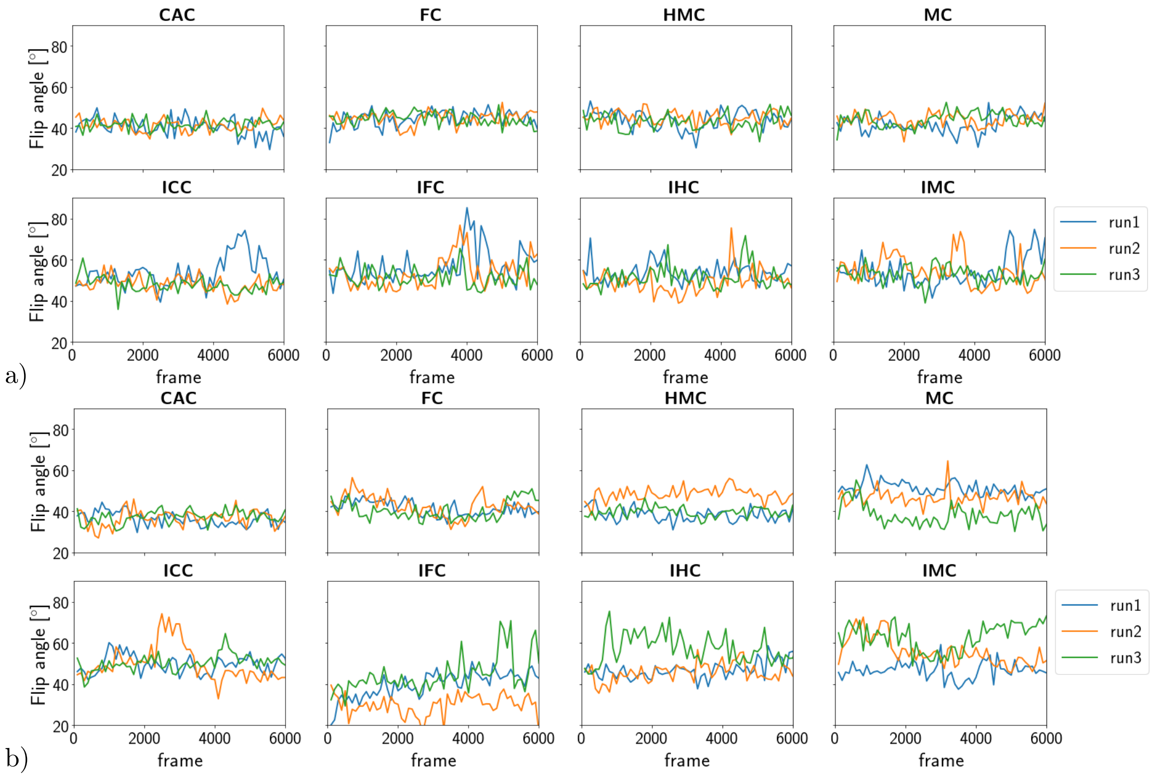

Figure S5: Time series of the flip angle in the individual runs of a) free DNA and b) DNA complexed to TDG.

Hydrogen Bonds

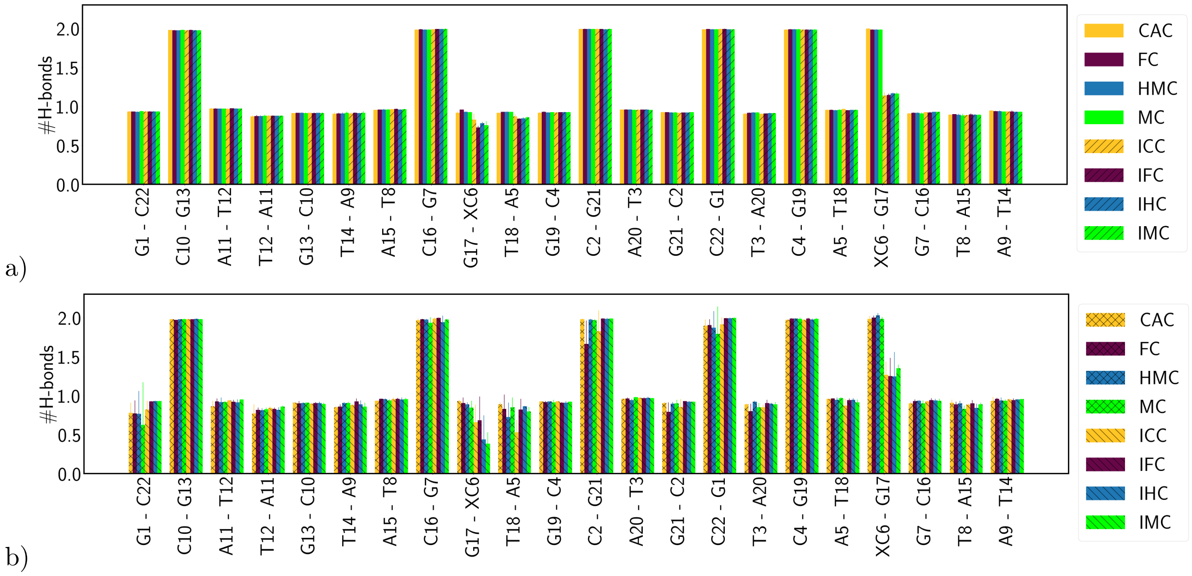

Figure S6: Hydrogen bond probabilities between DNA base pairs, other than the XC:G pair, in a) the free DNA and b) the DNA complexed to TDG. Note more than one hydrogen bond can be formed simultaneously.

Protein-DNA interactions

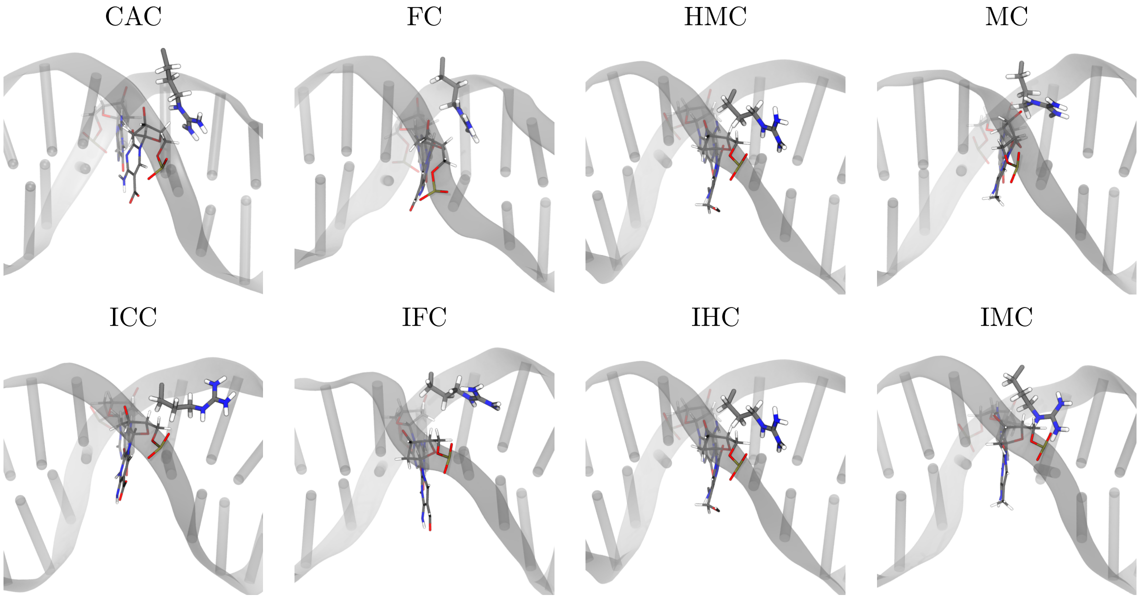

Figure S7: Snapshots of the DNA carrying XC:G complexed to TDG, showing the intercalated ARG275.

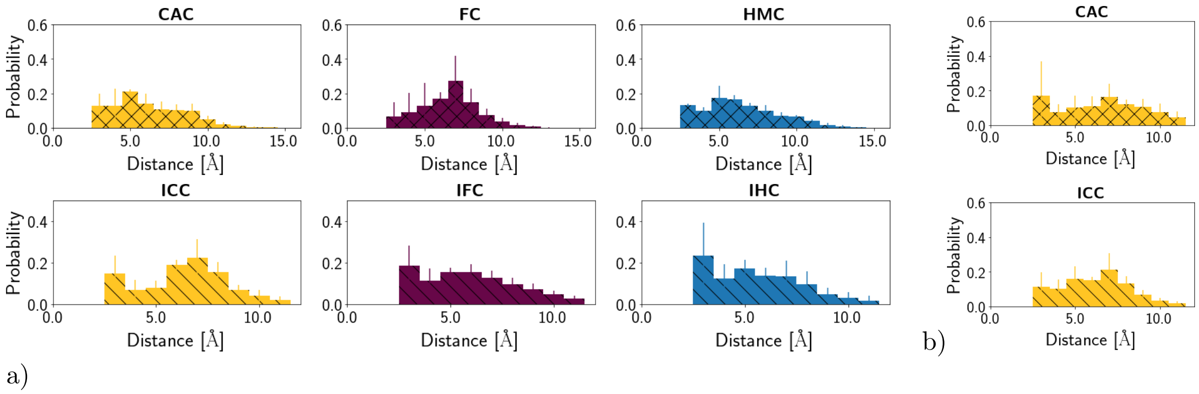

Figure S8: Probability distribution of the distances between Lys201 (NZ atom) and a) the O15 atom of the of the XC base and b) the O16 atom of the carboxyl group of the ICC.

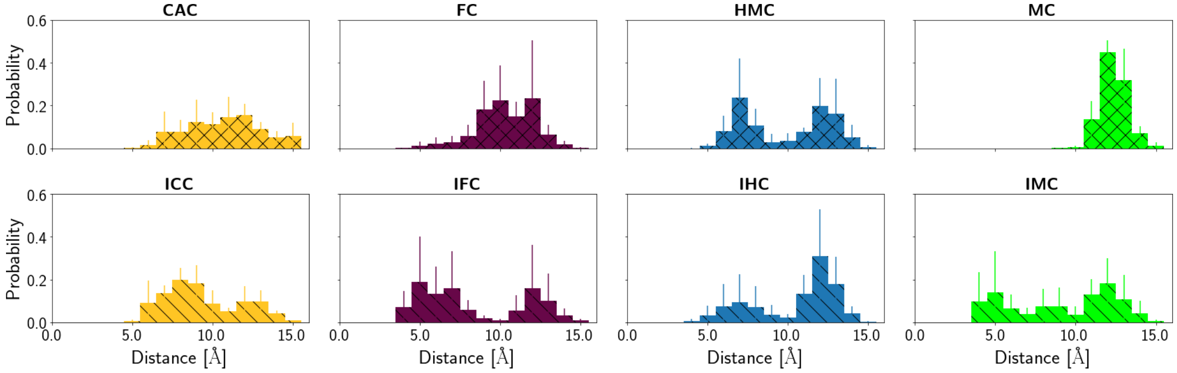

Figure S9: Probability distribution of the distances between the CB atom of Pro202 and the methyl carbon atom of the XC base.

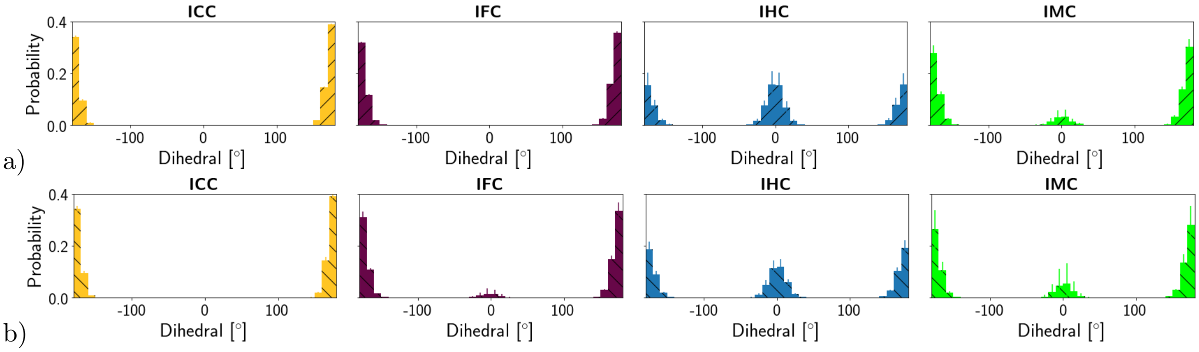

Figure S10: Probability distribution of the dihedral angle formed by atoms NH41-N4-C4-N3 of the XC base in a) free DNA and b) DNA complexed to TDG.
